# Influence of Compliance to Diet and Self-Efficacy Expectation on Quality of Life in Patients with Celiac Disease in Spain

**DOI:** 10.3390/nu12092672

**Published:** 2020-09-02

**Authors:** Ricardo Fueyo-Díaz, Miguel Montoro, Rosa Magallón-Botaya, Santiago Gascón-Santos, Ángela Asensio-Martínez, Guillermo Palacios-Navarro, Juan J. Sebastián-Domingo

**Affiliations:** 1 Department of Psychology and Sociology, Universidad de Zaragoza, 50009 Zaragoza, Spain; sgascon@unizar.es (S.G.-S.); acasensi@unizar.es (Á.A.-M.); 2 Aragon Institute of Health Science IACS, 50009 Zaragoza, Spain; maimontoro@gmail.com (M.M.); jjsebast@unizar.es (J.J.S.-D.); 3 Aragon Health Research Institute IISA, Group B21-17R, 50009 Zaragoza, Spain; rosamaga@unizar.es; 4 RediAPP Primary Care Prevention and Promotion Network RD16/07/05, Institute of Health Carlos III, 28029 Madrid, Spain; 5 Unit of Gastroenterology, Hepatology and Nutrition, Hospital General San Jorge, 22004 Huesca, Spain; 6 Department of Medicine Psychiatry and Dermatology, University of Zaragoza, 50009 Zaragoza, Spain; 7 Department of Electronic Engineering and Communications, University of Zaragoza, 50018 Zaragoza, Spain; guillermo.palacios@unizar.es; 8 Department of Digestive Diseases, Hospital Royo Villanova, 50015 Zaragoza, Spain

**Keywords:** celiac disease, self-efficacy, adherence to GFD, perceived HRQoL

## Abstract

The purpose of this study is to understand the health-related quality of life (HRQoL) in patients with celiac disease (CD) and analyze its main determinants. A transversal descriptive study of 738 patients with celiac disease was carried out. A series of questionnaires were answered related to their HRQoL, adherence to a gluten-free diet (GFD), and self-efficacy beliefs among other relevant variables. Regression analyses were carried out in order to explore the predictive variables in adherence to the GFD and HRQoL. A total of 61.2% showed a good HRQoL, and the main predictors of HRQoL were specific self-efficacy, adherence to the diet, risk perception, time since diagnosis, and age. While 68.7% of participants showed good or excellent adherence to the GFD, and the main predictors of adherence were specific self-efficacy, perceived adoption of recommended behaviors, HRQoL and gender. The HRQoL of patients with CD, and adherence to the GFD in Spain, are good. It is the self-efficacy expectation, measured specifically and not generally, which is the best predictor of both adherence and HRQoL. It is necessary to develop programs to improve the HRQoL of patients with CD that focus on improving specific self-efficacy.

## 1. Introduction

In the last decades, health-related quality of life (HRQoL) has become an important issue in research. HRQol can be defined as “how well a person functions in their life and his or her perceived wellbeing in physical, mental, and social domains of health” [1]. Still, there is a growing interest in clarifying this term as well as in identifying the factors that affect HRQoL, in order to design more effective and efficient treatments for any chronic disease [2,3].

Celiac disease (CD) is a common systemic disease, mediated by immune function, which manifests as an enteropathy of the small intestine, from exposure to gluten [4] present in wheat, barley, rye, and some types of oats [5]. Its prevalence in Europe is 1% in both children and adults [6]. However, the variety of related clinical symptoms which may appear makes it difficult to diagnose and it is estimated that an under diagnosis of 1:7 may exist [7]. Prevalence studies indicate a 2:1 female:male ratio [8]. In Spain, epidemiological studies are scarce and with small samples sizes, with percentages ranging from 0.23% to 0.85% [9,10,11,12,13,14]. More recently, we have found a prevalence of 0.35% in Aragon [15]. CD is considered a systemic disease that, if left untreated, can lead to a wide range of gastrointestinal and nutritional problems such as osteoporosis, infertility, or some types of lymphoma [8]. Currently, the only treatment identified for the disease is a strict lifelong gluten-free diet (GFD).

Despite the benefits of this GFD, adherence to it is far from perfect. Different studies place this adherence in a wide range (36–90%, with a median of 70%) [16], depending on how we define “strict adherence” and the type of measures considered. In Spain, studies have found adherence rates to be between 72% and 83% [17,18,19]. This adherence to the GFD is fundamental since 30% of the cases where no improvement in CD is seen, seem to be related to continuous exposure to gluten in the diet [20,21]. The persistence of symptoms, despite a GFD, leads to a reduce HRQoL, especially in the emotional sphere [22].

Several factors may play an important role in adherence to GFD, such as the cost of specific products, concern about the risk of intentional or accidental exposure to gluten, knowledge of what a GFD is, the ability to follow the diet when traveling, eating out, and at social events, belonging to a patient association, being comfortable with the GFD at work, belief in its importance to health, or changes in mood or stress [23]. Other studies have shown the importance of self-efficacy beliefs, when measured in a specific way in the adherence to the GFD [24,25,26,27]. Within social cognitive theory, self-efficacy is defined as beliefs in one’s capabilities to organize and execute the action required to achieve given goals [28]. It is in this context where we study self-efficacy expectations: the belief in patients with CD in organize and execute the actions required to adhere to their GFD in different situations.

Different qualitative studies [29,30,31] have investigated the difficulties that people diagnosed with CD have to face in everyday life. These studies identified that they experience problems in five areas: (1) shopping: the patient with CD has difficulties reading the labelling or dealing with possible changes in the formulations of the products they usually purchase; (2) travel problems such as finding a GFD offering in another city or managing their diet in a different language; (3) challenges at work or in their studies, such as the lack of any provision of GFD options, as well as difficulties in participating in school activities and work events; (4) eating with others at home: where they are forced to correct unsafe behavior by others or avoid appearing rude with food that others have provided; (5) eating out: identifying themselves publicly as a person with CD, requesting a gluten-free menu, having an unsafe dish removed or taking out food that they have brought from home, etc., are other difficulties that a person with CD has to face.

Consequently, emotions such as isolation, shame, fear of being contaminated by gluten or not wanting to cause discomfort are common among these patients. These feelings are sometimes the cause, and on other occasions the consequence, of the restrictions on their choices, which has the effect of producing an increased workload, or the need to be constantly alert, and a source of stress [29,30,31].

Partly, because of the lack of validated and cross-culturally adapted instruments, few studies have been carried out in Spain to analyze the HRQoL of patients with CD [32]. The perceptions of social isolation or lack of awareness of the disease by society have been reported as thoughts associated with the diagnosis of CD [33]. Other factors identified that influence the HRQoL of patients with CD in Spain are the quality of adherence to the GFD, absence of symptoms [18], time since diagnosis, and time on a GFD [34].

With this research, we aim to study the adherence to the GFD and HRQoL in patients with CD in Spain. We hypothesize that patients with CD with higher self-efficacy expectations will have better adherence to GFD and, therefore, a better HRQoL.

## 2. Materials and Methods

The study population was composed of patients residing in Spain. The inclusion criteria were being 18 years of age or older, having a medical diagnosis of CD, and being prescribed a strict lifelong GFD. These criteria were determined by answering three self-reported questions in the affirmative.

Based on our previous research [27], patients included in the study completed a 190 item questionnaire regarding sociodemographic data (age, gender, place of residency, education, occupational status), clinical data (previous symptoms and their intensity, age at diagnosis, time with symptoms before diagnosis, time since diagnosis), and GFD related issues (associated feelings, experiences, and perceptions), and scales regarding HRQoL, adherence to the GFD, and expectations of self-efficacy. Participants also answered questions regarding the perceived adoption of recommended behaviors and their perception of the risks associated with not following those recommendations.

The patients were recruited online through the regional patients’ associations in Spain and through associations’ social networks during September–November 2019 and invited to complete the questionnaires online using Google Forms (Google, Mountain View, CA, USA). After receiving written information about the study through the regional associations, patients signed an informed consent document. The program SPSS v21 (IBM, Armonk, NY, USA) was used to perform the statistical analysis. Absolute frequencies were used in the descriptive analysis while relative frequencies were used for the qualitative analysis. Pearson’s chi-squared was used for gender in the bivariate analyses, while for quantitative variables a t-student test was used. Age group comparisons were performed by one-way ANOVA and post hoc evaluations by Scheffe’s test. Regression analyses were carried out to study the relationship between the dependent variable, HRQoL, and the independent variables of adherence to the GFD, and the other variables. In another regression analysis, adherence to the GFD was considered as a dependent variable and the rest of the factors studied as independent variables. The statistical significance was set at *p* < 0.05. This study was approved by the Ethics Committee of Clinical Research of Aragon (CEICA) with number CIPI16/0311.

### 2.1. Adherence to the GFD

Adherence to the GFD was evaluated with the celiac dietary adherence test (CDAT) questionnaire [35]. This questionnaire consists of 7 questions. It is easy to apply and has good psychometric properties. It has the advantage of being correlated with serological and histological variables as well as with interviews with nutritionists. The questionnaire was validated in Spain and again presented good psychometric properties [19]. Patients have to answer on a 5-point scale. The scores are additive (7–35), with higher scores indicating lower adherence. Scores below 13 indicate excellent or very good adherence, 13–17 moderate adherence, while scores above 17 indicate fair or poor adherence [35]. We added a separate question regarding adherence, where participants were asked to rate their adherence on a 0–10 scale, considering strict adherence to GFD scores ≥ 9.

Finally, according to the CDAT cut-off point, participants were divided into a moderate/low adherence group (group 1, with scores ≥ 13) and an excellent/good adherence group (group 2, with scores < 13). We compared differences between these two groups for the variables of the study. We also divided the participants into 3 age groups to compare differences.

### 2.2. General and Specific Self-Efficacy

General self-efficacy was evaluated with the Spanish adaptation of the general self-efficacy scale (GSES) [36]. This scale is comprised of 10 items (e.g., “I can solve difficult problems if I make enough effort”) to which the patient answers on a 4-point Likert scale (1 = not at all true, 2 = hardly true, 3 = moderately true, 4 = exactly true). The scores are additive in a range of 10 to 40, with the highest scores indicating greater self-efficacy. Although the authors do not set a cut-off point, we will consider the 70% of the theoretical rank (score ≥ 31) as indicative of high general self-efficacy [37,38].

CD specific self-efficacy was evaluated using the celiac disease self-efficacy scale (Celiac-SE) [39] designed to measure the degree of perceived self-efficacy in adherence to the GFD in patients aged 12 years or older, in different situations such as shopping, eating at home with others, traveling, eating out, or eating at work or school. Patients answered a 25-question questionnaire between 0 (I could definitely not do this) and 10 (I am completely certain I could do this). The questionnaire provides an average score for the whole scale and for each of the areas. Scores at 7 and above indicate high self-efficacy. Cronbach’s alpha for the total scale is 0.81.

### 2.3. HRQoL

The HRQoL of patients with CD was evaluated using the Spanish version of the CD Quality of life survey (CD-Qol) [32]. This questionnaire consists of 20 questions that are answered on a 5-point Likert scale. The questions are grouped into four dimensions: limitations, dysphoria, health concerns, and inadequate treatment. The scores are additive in a range of 10 to 100. Scores of 70 or higher indicate good HRQoL.

It is important to mention that we found a discrepancy in the wording of item 8, and we communicated this issue to the authors. Since all responses were considered to be additive in this questionnaire, the correct wording of this item must be negative (“I feel that diet is not sufficient treatment for my disease”). We believe that this was the original intention, but that the word “not” was accidentally omitted in the version of the questionnaire that was published in the appendix of Dorn et al.’s 2010 validation paper. This mistake can also be found in the Spanish adaptation. This item affects the subscale “inadequate treatment”.

In order to compare HRQoL with studies of other diseases, participants responded to the Spanish version of the SF 12-item Short Form Health Survey (SF-12) [40]. It is an adaptation of the SF-36 which generates a physical component summary (PCS) and a mental one (MCS).

### 2.4. Perception of Risk, Perception of Adoption of Recommended Behaviors, Time Since Diagnosis, Perceived Consequences of Abandoning the Diet, Presence of Digestive and Non-Digestive Symptoms, and Presence of Associated Diseases

Additionally, we evaluated, using two questionnaires, the perception of risks and the perceived adoption of the behaviors recommended when dealing with the GFD. These two questionnaires were developed from the content in the patients’ association guide which was elaborated by experts [41], and previously used in our research [27,42]. The first questionnaire was the celiac disease risk assessment questionnaire (CDRAQ). This was an 18-item questionnaire, scored from 0 (not risky) to 10 (very risky), according to a patient´s perceived risk with respect to certain behaviors. The scores are additive, with higher scores indicating a stronger perception of risk. Examples of these items are: “I consider it to be a risk to consume processed products without a gluten free label” or “not eliminating bulk products from diet”. In the present study this questionnaire showed a Cronbach’s alpha of 0.91.

The second questionnaire was the celiac disease recommended behaviors questionnaire (CDRBQ). This second questionnaire consists of 18 questions where participants must assess if they follow the recommended behaviors, rated 0 (I do not follow this recommended behavior) to 10 (I follow this recommended behavior). An example of these items is “I wash my hands after touching something with gluten”. Scores are additive and higher scores indicate stronger perception of the correct adoption of the recommended behaviors. In this study this questionnaire showed a Cronbach’s alpha of 0.81.

Other variables studied were the time since diagnosis, the perceived consequences of abandoning the diet, the presence or not of digestive or non-digestive symptoms, and the presence of associated diseases.

### 2.5. Perceptions Associated with CD

We also included the celiac disease perceptions and emotions questionnaire (CDPEQ) to evaluate the frequency of some perceptions and emotions in these patients. This questionnaire was developed by this research team from the results of qualitative studies [29,31], and used previously in our research [42] to explore if those feelings and perceptions are common among Spanish patients with CD. This is a 17-item questionnaire that had to be answered on a 5-point Likert scale (never, sometimes, average, many times, always). The items asked included how often the patient feels that they have an unwanted role in social events, feel rejected or feel ignored by others, and questions about the frequency with which the patient experiences feelings of anxiety or sadness related to the illness.

### 2.6. Socio-Demographic Variables

Several sociodemographic questions related to age, place of residence, marital status, work, or educational level were incorporated.

## 3. Results

A total of 818 questionnaires were collected, of which 738 were from patients with CD, 45 reported non-celiac gluten sensitivity, 14 were from minors, 7 had consent problems, and 14 were incomplete.

### 3.1. Description of the Sample

Of the 738 patients with CD (85.3% women), 78.2% belonged to a patient association. The age range was between 18 years and 74 years (M = 39.41; SD = 11.57). Most participants had several years of experience with the disease (M = 9.80; SD = 10.12). Table 1 below displays the main characteristics of the sample.

### 3.2. Adherence to GFD

In all 96.88% of the participants reported a strict adherence to GFD when asked in the separate question added to the CDAT. The CDAT results showed good adherence (M = 11.49, SD = 2.97) in Table 2. In addition, 68.7% showed good or excellent adherence, with scores below 13 while 4.6% reported poor adherence with scores above 17. We found better adherence in those above 35 years of age (*p* < 0.001). Gender differences were found with women showing greater adherence (*p* = 0.012). 

### 3.3. Self-Efficacy

Participants showed high general self-efficacy (M = 31.34; SD = 4.84) and we found differences between the high and moderate/low self-efficacy groups (*p* < 0.001). In relation to specific self-efficacy, this was high (M = 8.68; SD = 1.20) and we found differences between the groups of high and moderate/low adherence, with participants with better adherence to the GFD showing higher specific self-efficacy (*p* < 0.001).

In a more detailed analysis (Table 3), we found that the lowest self-efficacy occurs in the area of “traveling” (M = 7.95; SD = 1.83), while the highest self-efficacy appears in the area of “eating at home with others” (M = 9.37; SD = 1.47). Here, we have also found differences between the excellent/good and moderate/low adherence groups (*p* < 0.01) for each of the areas. No gender differences were found for specific self-efficacy. We found significant differences between groups (*p* < 0.001). The self-efficacy levels in the 18–35 group were significantly lower (*p* < 0.001) than for the 36–50 group. No differences were found between 36–50 and the >50 groups (*p* = 0.069).

If we set a cut-off point of seven where lower scores indicate low/moderate self-efficacy, 9.7% have low/moderate specific self-efficacy for managing a GFD. In an area analysis, 25.9% show low/moderate specific self-efficacy for “traveling”, 15.5% for “eating at work or school”, 11.4% for “eating out”, 8.4% for “shopping”, and 7.3% for “eating at home with others”.

### 3.4. HRQoL

The results in HRQoL with the SF12v2 questionnaire were moderately high (M = 36.59; SD = 4.47) with the physical component (M = 16.29; SD = 2.02) being slightly lower than the mental one (M = 20.30; SD = 3.06) (Table 4). If we move it to a scale of 0 (worst HRQoL) to 100 (best HRQoL) we get a 62.65 (physical), 67.67 (mental) and 65.34 for the overall scale. We find differences in the adherence groups for MCS (*p* < 0.001) but not for the PCS. No gender differences were found in any of the dimensions.

The results relating to HRQoL measured with CDQol were high (M = 72.73; SD = 16.83). If we express the four dimensions on a scale of 0–100, “health concerns” with a 66.92% (M = 16.73; SD = 5.16) had the lowest perceived HRQoL, followed by “limitations” with 70% (M = 31.50; SD = 9.10), “inadequate treatment” with 72.70 (M = 7.27; SD = 2.28), and “dysphoria” with 86.15% (M = 17.23; SD = 3.16). Additionally, 38.8% of patients obtained a score below 70, the cut-off point above which the score indicates good HRQoL.

Significant differences in HRQol in CDQoL were found between the high and moderate/low adherence groups (*p* < 0.001), with higher scores for those who had better adherence to the GFD. No gender differences were found. It is the over 50 age group which shows a better HRQoL, with significant differences with the other two groups (*p* < 0.001), while the 18–35 group does not reach the cut-off point of 70, indicative of high HRQoL.

### 3.5. Perception of Risk (CDRAQ), Perception of Adoption of Recommended Behaviors (CDRBQ), Age at Diagnosis, Time Since Diagnosis, Intensity of Symptoms, and Consequences of Abandoning

The excellent/good adherence group had a higher risk perception (*p* < 0.038) and reported a higher follow-up of recommended behaviors (*p* < 0.001) (Table 5). Differences between high and low/moderate adherence groups were found (*p* < 0.047) for consequences of abandoning the GFD. Paradoxically, the low adherence group perceived the consequences of abandoning the diet to be more serious. Furthermore, differences in symptom intensity after transgression were found to be higher in the moderate/low adherence group (*p* = 0.006). Age at diagnosis (*p* = 0.299) and time since diagnosis (*p* = 0.056) do not seem to play a major role in adherence to the GFD.

### 3.6. Relationship between the Variables, GFD and HRQoL

Table 6 shows the results of the linear regression to study the impact of changes in the independent variables on adherence to the GFD. After studying the main variables, we only show those that have a very clear effect: specific self-efficacy, perceived adoption of patient association recommendations, risk perception, HRQoL (when measured with CDQoL), and gender, which are all significant. The model with these five variables explains 25.9% of the variance in GFD adherence. In relation to the semi-partial correlations, the specific self-efficacy represents 6.7%, while the HRQoL represents 5.1%. This would mean that the higher the specific self-efficacy, HRQoL, and the perception of adherence, the higher the adherence to a GFD, as the perception of risks does not appear to have an effect on the adherence to a GFD. Being a woman is also a predictor of adherence.

Age, age at diagnosis, time since diagnosis, intensity of symptoms, risk perception, or belief in the serious consequences of abandonment were not predictive of adherence.

If we analyze HRQoL as a dependent variable (Table 7), this is explained by specific self-efficacy, adherence to the GFD, time since diagnosis, age and risk perception, with the perceived adoption of the recommended behaviors not being significant. In this model, these variables explain 30.6% of the HRQoL score. As far as semi-partial correlations are concerned, the specific self-efficacy variable explains 6.2% of the variability in HRQoL, 5.5% the time since diagnosis, while adherence to the GFD, explains 4% of the variability, while risk and age, although significant, have a negligible influence, 2.6% and 1.5%, respectively. No differences based on gender are found, neither for the questionnaire as a whole nor for each of the scales.

### 3.7. Perceptions and Emotions Associated with Having CD, According to CDPEQ

Lastly, participants were also asked about their perceptions and emotions associated with having CD (Table 8). The most common perceptions were of a limited gluten-free offering (68.6%), the feeling of having to be constantly alert (60.7%), or an excessive protagonism in social events (55.9%). Also noteworthy is the perception that they have to work twice as hard because of their illness (shopping, cooking, etc.). It is also common to have the feeling that they are forgotten about at social events (31.2%). With regard to the most frequent negative emotions, anger (25.2%), envy (21.4), sadness (17.2%), anxiety (14.2%), or fear (13.9%) stand out, but it is worth noting the high frequency of positive feelings associated with the disease such as pride and self-confidence (50.5%), or joy (30.9%).

## 4. Discussion

This research shows that specific self-efficacy, adherence to the GFD as well as risk perception, time since diagnosis, and age play an important role in the HRQoL of patients with CD. Results show that it is self-efficacy, when specifically measured, that best predicts adherence to the GFD and the resulting HRQoL [27,39].

### 4.1. Adherence to GFD

Adherence to a GFD was high in this study, with 68.7% of participants showing good or excellent adherence according to CDAT criteria [35], but somewhat lower than in previous research [27] conducted in Spain. This high adherence may be due to the fact that a large part of the sample (78.2%) is associated with a patient association [16,23,43] and, therefore, has a high level of knowledge about the disease with almost 10 years of average experience of the GFD. In our study, this adherence to a GFD is determined by specific self-efficacy, HRQoL (measured with CDQoL) and perceived adoption of recommended behaviors, with women showing better adherence than men. Patients above 35 years old also show a better adherence than younger individuals.

We should note that it is self-efficacy measured in a specific way, rather than general self-efficacy, that plays a predictive role in adherence to the GFD [27,44]. Risk perception, although not a significant predictor, is included in the model because of the role it can play in the development of self-efficacy in adherence to risk, as people with high self-efficacy perceive risks as challenges, while people with low self-efficacy may perceive them as overwhelming threats [28].

No significant relationships were found with time-related variables such as age, age at diagnosis, or time since diagnosis [27], despite the predictive value that other studies place on age [24,45] or age at diagnosis [45].

Determining the degree of adherence to the GFD is complex, varies from study to study [16], and often involves subjective responses to self-reported questions (in our study, 96.88% reported strict adherence, well above the 68.7% indicated by CDAT). This subjective perception of adherence may be conditioned by their knowledge of the disease, its consequences, and an adequate or inadequate risk assessment. It is clear that there is a need to develop new tools to assess adherence as current histological and serological methodologies are expensive and invasive and not very sensitive to the detection of transgressions. Evaluations by expert dietitians [46,47] and the detection of immunogenic peptides [48,49] have been shown to be effective in the follow-up of patients with CD. Even so, there is a need for the development of new questionnaires for the assessment of adherence to GFD that take into account variables such as knowledge about the disease and the behaviors that determine adherence, the expectation of specific self-efficacy, and risk assessment.

### 4.2. Self-Efficacy

General self-efficacy levels were significantly higher compared to other studies [27,50,51,52]. Patients with CD may develop these high levels because they have to manage the disease and its treatment for years, unlike the case of other diseases where that responsibility lies more with the doctor [53]. We also think that this responsibility in managing one’s own disease and the GFD can contribute to this general feeling of self-efficacy. The same occurs with the level of specific self-efficacy which is high, and it is comparable to other studies recently carried out in Spain [27]. Considering regression analysis, it seems that specific rather than general self-efficacy plays a role in predicting adherence to a GFD, which is in line with previous research [27], and which emphasizes the need to measure control beliefs more specifically rather than in a general way. The results in our study support the idea, as in other studies [27,29,39], that the areas in which people with CD are less confident are “traveling,” “shopping,” and “eating out”.

Neither in specific self-efficacy, nor in general, were differences found based on gender, time on a GFD, or year of diagnosis, which would emphasize the idea that it is more the type of situation and its management than other personal characteristics (such as experience with the disease) which explain the development of self-efficacy beliefs [28]. In a more detailed analysis by age, it is above 35 years old where we find a higher sense of specific self-efficacy. The fact that differences are found between groups, with different levels of adherence between the different areas evaluated by the Celiac-SE, reinforces the idea of the importance of the situation, and that we must have instruments that allow us to evaluate self-efficacy, and that it is useful to do so for each of the areas where difficulties may appear in the management of the GFD. As such, in a newly diagnosed patient, we can determine their levels of self-efficacy in “traveling” or “eating at work” which may well be different, and need different types of support to function in one area or other. This evaluation of specific self-efficacy with respect to each area of CD management will allow us to design tailored interventions in the clinical setting to improve adherence. Celiac-SE is a useful instrument for this purpose.

### 4.3. HRQoL

The results of this study show good HRQoL, although 38.8% of the participants showed a low HRQoL below the cut-off point of 70 that is established as indicative of a good HRQoL [32]. It is “health concerns” that have the lowest score as in previous studies [27,32]. These studies disagree with one recently published, with a very large sample (*n* = 1230), where the overall score was significantly lower (56.3 ± 18.27) but where, as in our study, “health concerns” [34] showed one of the lowest scores.

According to our model, HRQoL is better when specific self-efficacy expectations are higher, the adherence is better, the risk perception is lower, and the age and time from diagnosis is higher. It is self-efficacy, again measured in a specific, rather than a general way, that best explains, of these factors, the HRQoL score [27]. Good adherence, among other factors due to the elimination of symptoms and health problems, is a good predictor of HRQoL [18,54]. A low perception of the risks associated with the disease, coupled with high self-efficacy [24,44], predict a better HRQoL. Finally, a broader experience with the disease and with the GFD can also lead to a better HRQoL [34]. Age is significant for the questionnaire as a whole and for each of the subscales, but with low correlations, while recent studies in Spain do not find this relationship except for the “health problems” scale [34]. In our study it is participants above 50 years old that show a better HRQoL.

In terms of the associated feelings and emotions, it seems that patients with CD have a feeling of having to be alert, constantly watching their diet, and that it is uncomfortable for them to take an unwanted prominence in celebrations involving food. These and other circumstances often generate feelings of anger or envy in one-fifth of them. Feelings of anxiety or fear, which are more directly mediated by their high expectations of self-efficacy, are more subdued, the latter not being as frequent. These feelings are in line with other research [29,30,31,34,42]. Remarkably, we did identify in this study feelings of pride or joy associated with the disease quite often which may indicate a correct acceptance of the diagnosis. This may be explained by the fact that the patients have a lot of experience with GFD and that most of them belong to patient associations. Finally, 43.2% of the participants reported associated diseases which may have affected their HRQoL.

This study has several limitations: first, participants were recruited mainly through patient associations so there may be an under-representation of patients with CD who are not associates. Second, although an interview with an expert dietician is usually required to become an associate, this is not always the case and participants were included by means of questions in which they stated that they had a firm diagnosis of CD and that they were, therefore, prescribed a lifelong GFD. On the other hand, although the sample is large, it was not possible to obtain a sufficiently representative sample of the Spanish population. Moreover, we must be cautious when interpreting gender differences as 85.3% of the respondents were women and this may affect the analysis. Finally, as it is a long survey, this could have influenced the number of questionnaires obtained. As strengths of this study we must mention that this research explores the HRQoL in patients with CD in Spain, their feelings and expectations with a substantial sample and highlights the variables to be included in programs for the management of the disease by patients with CD. This type of study is not frequent in Spain. Secondly, we found the discrepancy in item eight of the English version of the CD-Qol questionnaire and, at least, the Spanish version. The Italian version seems to be correct. We have communicated this issue to the authors, who have responded that they are going to adopt the necessary measures to solve this problem. This questionnaire has been widely used in research in CD [27,32,34,42,55,56].

In recent years, we have a clearer picture of what the characteristics are of patients with CD in Spain. The various studies indicate that they are mainly women, diagnosed in the first or fourth decade, belonging to patient associations, with high specific self-efficacy, good adherence to the GFD, and a good HRQoL. Even so, given that the only treatment to date for this disease is strict adherence to the GFD, specific programs must be developed in primary care and in patient associations themselves to care for these patients. In order to improve HRQoL in patients with CD, these programs should include, not only information on the disease, on the GFD and its risks but also an assessment of specific self-efficacy for each of the identified areas (shopping, traveling, eating out and at home with others and at work) and strategies to develop a strong sense of self-efficacy to manage the disease in these situations.

## 5. Conclusions

In Spain, people with CD have a good adherence to the GFD and a good HRQoL. The expectation of self-efficacy, measured in a specific rather than a general way, is the best predictor of both adherence and subsequent HRQoL. New GFD adherence programs for patients with CD must assess and promote the expectation of specific self-efficacy for each of the areas in which these patients have to achieve adherence.

## Figures and Tables

**Table 1:** Sample characteristics.

Characteristic	*n* = 738
Mean age ± standard deviation (years)	39.41 ± 11.57
Age groups 18–35/36–50/>50	181/427/126
Gender (% female)	85.3
Mean age at diagnosis ± standard deviation (years)	30.78 ± 15.59
Mean time since diagnosis ± standard deviation (years)	9.80 ± 10.12
Associated to a support group (%)	78.2
Nationality (% Spanish)	94.3
Civil status (% married/single/divorced/other)	68.97/26.96/1.90/2.17
Self-reported gluten-free diet (GFD) adherence (% strict)	96.88
Intensity of symptoms after transgressions (%none/mild/moderate/intense/very intense)	22.29/18.23/22/24.17/13.31
Associated diseases (%)	43.2
Thyroid problems/food intolerance/allergies/asthma/diabetes/other (%)	8.81/4.61/3.79/3.12/2.44/77.24
Education (% primary/secondary/university/other)	2.2/23.2/74.1/0.5
Years with symptoms before diagnosis % (0/<1/1–5/>5)	7.46/20.17/32.87/39.50
Presence of digestive symptoms before diagnosis (%)	72.5
Presence of non-digestive symptoms before diagnosis (%)	63.0

**Table 2:** CDAT total scores and divided according to age (*n* = 738).

Variable	*n*	Mean	SD	*p*
Adherence to GFD *	738	11.49	2.97	
18–35 years	181	12.12	3.31	*p* < 0.001
36–50 years	427	11.42	2.76	
>50 years	128	10.84	2.93	

* Scores below 13 indicate high adherence. CDAT: celiac dietary adherence test. GFD: Gluten Free Diet.

**Table 3:** General and specific self-efficacy by adherence group, age group, and area of Celiac-SE (*n* = 738).

Variable	*n*	Mean	SD	*p*
General self-efficacy (GSES) *		31.34	4.84	
Group 1	231	30.04	4.71	<0.001
Group 2	507	31.93	4.78	
Specific self-efficacy (Celiac-SE) **		8.68	1.20	
Group 1	230	8.07	1.36	<0.001
Group 2	506	8.96	0.04	
18–35 years	181	8.28	1.30	<0.001
36–50 years	427	8.75	1.15	
>50 years	126	9.02	1.07	
Shopping		8.65	1.20	
Group 1	231	8.06	1.31	<0.001
Group 2	507	8.92	1.04	
Traveling		7.95	1.83	
Group 1	231	7.22	2.03	<0.001
Group 2	507	8.28	1.63	
Eating at home with others		9.37	1.47	
Group 1	231	9.01	0.67	<0.001
Group 2	507	9.54	1.33	
Eating outside with others		8.73	1.64	
Group 1	231	8.04	1.94	<0.001
Group 2	507	9.05	1.37	
At work or Studies		8.70	1.87	
Group 1	230	7.98	2.17	<0.001
Group 2	506	9.03	1.62	

Group 1 = moderate/low adherence group with CDAT scores ≥ 13; Group 2 = excellent/good adherence group with CDAT scores < 13. * Scores at and above 31 indicate high general self-efficacy. ** Scores at 7 and above indicate high specific self-efficacy. GSES: general self-efficacy scale; Celiac-SE: celiac disease self-efficacy scale. CDAT: celiac dietary adherence test.

**Table 4:** Health-related quality of life (HRQoL), according to SF-12 and CDQol results, by adherence and age group (*n* = 738).

Variable	*n*	Mean	SD	*p*
SF-12 overall score		36.59	4.47	
Physical (PCS)		16.29	2.02	
Group 1	231	16.23	2.42	0.540
Group 2	507	16.32	1.81	
Mental (MCS)		20.30	3.06	
Group 1	231	18.97	3.31	<0.001
Group 2	507	20.91	2.73	
CDQol overall score *		72.73	16.83	
Group 1	228	64.64	17.03	<0.001
Group 2	506	76.36	15.43	
18–35 years	178	69.81	17.31	<0.001
36–50 years	426	72.02	16.72	
>50 years	128	79.07	15.05	

Group 1 = moderate/low adherence group with CDAT scores ≥ 13; Group 2 = excellent/good adherence group with CDAT scores < 13. * Scores above 70 indicate high HRQoL. CDQoL: CD quality of life survey; SF-12: SF 12-item short form health survey.

**Table 5:** Scores for risk perception (CDRAQ), perceived adoption of recommended behaviors (CDRBQ), age at diagnosis, time since diagnosis, intensity of symptoms, and consequences of abandoning.

Variable	*n*	Mean	SD	*p*
Risks perception		157.74	22.44	
Group 1	221	155.03	24.29	0.038
Group 2	487	158.98	21.47	
Perceived adoption of recommended behaviors		160.44	21.18	
Group 1	220	155.83	25.08	<0.001
Group 2	475	162.58	18.76	
Age at diagnosis		30.78	15.59	
Group 1	231	29.90	15.38	0.299
Group 2	507	31.19	15.68	
Time since diagnosis		9.80	0.12	
Group 1	224	8.72	9.59	0.056
Group 2	494	10.28	10.33	
Intensity of symptoms		2.88	1.36	
Group 1	217	3.09	1.30	0.006
Group 2	474	2.78	1.37	
Consequences of abandoning		2.27	0.54	
Group 1	228	2.33	0.58	0.047
Group 2	501	2.24	0.52	

Group 1 = moderate/low adherence group with CDAT scores ≥ 13; Group 2 = excellent/good adherence group with CDAT scores < 13. CDRAQ: celiac disease risk assessment questionnaire; CDRBQ: celiac disease recommended behaviors questionnaire.

**Table 6:** Linear regression analysis predicting GFD adherence.

Variable	B	β	R^2^	F
Specific self-efficacy	−0.741	−0.302 *	0.259	46.58 *
Recommended behaviors	−0.021	−0.152 *		
HRQoL	−0.044	−0.252 *		
Risk perception	0.007	0.052		
Gender	−0.789	−0.093 *		

* *p* < 0.01.

**Table 7:** Linear regression analysis predicting HRQoL.

Variable	B	β	R^2^	F
Specific self-efficacy	4.039	0.288 *	0.306	59.57 *
Risk perception	−0.129	−0.174 *		
GFD adherence	−1.281	−0.223 *		
Time since diagnosis	0.402	0.241 *		
Age	0.197	0.133 *		

* *p* < 0.01. GFD-gluten-free diet.

**Table 8:** Perceptions and emotions associated with having celiac disease (CD), according to CDPEQ.

Perceptions and Feelings	%
A perception that the gluten-free offering is restricted	68.6
Being constantly alert	60.7
Undesired protagonism in social events	55.9
Pride	50.5
You have to work twice as hard because you have CD	34.9
Joy	30.9
Being forgotten about	31.2
Anger	25.2
Envy	21.4
Fear	13.9
Shame	12.8
Sadness	17.2
Being rejected	11.5
Anxiety	14.2
Relief	11.5
An avoidance of mentioning that you have CD	6.1
Being obliged to take unnecessary risks	4.1

%: always or almost always. CDPEQ: celiac disease perceptions and emotions questionnaire.

## References

[1] Hays R., Reeve B., Killewo J., Heggenhougen H., Quah S. (2010). Measurement and modeling of health-related quality of life. Epidemiology and Demography in Public Health.

[2] Häuser W., Gold J., Stein J., Caspary W.F., Stallmach A. (2006). Health-related quality of life in adult coeliac disease in Germany: Results of a national survey. Eur. J. Gastroenterol. Hepatol..

[3] Karimi M., Brazier J. (2016). Health, Health-Related Quality of Life, and Quality of Life: What is the Difference?. PharmacoEconomics.

[4] Walker M.M., Ludvigsson J.F., Sanders D.S. (2017). Coeliac disease: Review of diagnosis and management. Med. J. Aust..

[5] Comino I., Real A., Moreno L., Lorenzo L., Cornell H., Lopez-Casado M., Barro F., Lorite P., Torres M., Cebolla A. (2011). Is it the oat a toxic cereal for coeliac patients? Its depends on the variety. Ann. Nutr. Metab..

[6] Ludvigsson J.F., Card T.R., Kaukinen K., Bai J., Zingone F., Sanders D.S., Murray J.A. (2015). Screening for celiac disease in the general population and in high-risk groups. United Eur. Gastroenterol. J..

[7] Farrell R., Kelly C. (2001). Diagnosis of celiac Sprue. Am. J. Gastroenterol..

[8] Gujral N. (2012). Celiac disease: Prevalence, diagnosis, pathogenesis and treatment. World J. Gastroenterol..

[9] Riestra S., Fernández E., Rodrigo L., Garcia S., Ocio G. (2000). Prevalence of Coeliac disease in the general population of northern Spain. Strategies of serologic screening. Scand. J. Gastroenterol..

[10] Castaño L., Blarduni E., Ortiz L., Núñez J., Bilbao J.R., Rica I., Martul P., Vitoria J.C. (2004). Prospective population screening for celiac disease: High prevalence in the first 3 years of life. J. Pediatr. Gastroenterol. Nutr..

[11] García Novo M.D., Garfia C., Acuña Quirós M.D., Asensio J., Zancada G., Barrio Gutierrez S., Manzanares J., Solís-Herruzo J.A. (2007). Prevalencia de la enfermedad celiaca en donantes de sangre de la Comunidad de Madrid. Rev. Esp. Enferm. Dig..

[12] Mariné M., Farre C., Alsina M., Vilar P., Cortijo M., Salas A., Fernández-Bañares F., Rosinach M., Santaolalla R., Loras C. (2011). The prevalence of coeliac disease is significantly higher in children compared with adults: Changing prevalence of coeliac disease in Catalonia. Aliment. Pharmacol. Ther..

[13] Cilleruelo M.L., Roman-Riechmann E., Sanchez-Valverde F., Donat E., Manuel-Ramos J., Martín-Orte E., López M.J., García-Novo D., García S., Pavón P. (2014). Spanish national registry of celiac disease: Incidence and clinical presentation. J. Pediatr. Gastroenterol. Nutr..

[14] Navalón-Ramon E., Juan-García Y., Pinzón-Rivadeneira A. (2016). Prevalencia y características de la enfermedad celíaca en la fachada mediterránea peninsular. Semer. Med. Fam..

[15] Fueyo-Díaz R., Magallón-Botaya R., Masluk B., Palacios-Navarro G., Asensio-Martínez A., Gascón-Santos S., Olivan-Blázquez B., Sebastián-Domingo J.J. (2019). Prevalence of celiac disease in primary care: The need for its own code. BMC Health Serv. Res..

[16] Hall N.J., Rubin G., Charnock A. (2009). Systematic review: Adherence to a gluten-free diet in adult patients with coeliac disease. Aliment. Pharmacol. Ther..

[17] Casellas F., Vivancos J.L., Malagelada J.R. (2006). Current epidemiology and accessibility to diet compliance in adult celiac disease. Rev. Esp. Enferm. Dig..

[18] Casellas F., Rodrigo L., Vivancos J.L., Riestra S., Pantiga C., Baudet J.S., Junquera F., Diví V.P., Abadia C., Papo M. (2008). Factors that impact health-related quality of life in adults with celiac disease: A multicenter study. World J. Gastroenterol..

[19] Fueyo Díaz R., Gascón Santos S., Asensio Martínez Á., Sánchez Calavera M.A., Magallón Botaya R. (2016). Transcultural adaptation and validation of the Celiac Dietary Adherence Test. A simple questionnaire to measure adherence to a gluten-free diet. Rev. Esp. Enferm. Dig..

[20] Leffler D.A., Dennis M., Hyett B., Kelly E., Schuppan D., Kelly C.P. (2007). Etiologies and Predictors of Diagnosis in Nonresponsive Celiac Disease. Clin. Gastroenterol. Hepatol..

[21] Syage J.A., Dickason M.A., Voyksner J.A.S. (2017). Celiac Disease Patients Practicing a Gluten-Free Diet Still Consume Unsafe Levels of Gluten. Gastroenterology.

[22] Harnett J.E., Myers S.P. (2020). Quality of life in people with ongoing symptoms of coeliac disease despite adherence to a strict gluten-free diet. Sci. Rep..

[23] Leffler D.A., Edwards-George J., Dennis M., Schuppan D., Cook F., Franko D.L., Blom-Hoffman J., Kelly C.P. (2008). Factors that influence adherence to a gluten-free diet in adults with celiac disease. Dig. Dis. Sci..

[24] Ford S., Howard R., Oyebode J. (2012). Psychosocial aspects of coeliac disease: A cross-sectional survey of a UK population. Br. J. Health Psychol..

[25] Sainsbury K., Mullan B., Sharpe L. (2012). Social-cognitive and psychological predictors of gluten free diet adherence in coeliac disease. Psychol. Health.

[26] Sainsbury K., Halmos E.P., Knowles S., Mullan B., Tye-Din J.A. (2018). Maintenance of a gluten free diet in coeliac disease: The roles of self-regulation, habit, psychological resources, motivation, support, and goal priority. Appetite.

[27] Fueyo-Díaz R., Magallón-Botaya R., Gascón-Santos S., Asensio-Martínez Á., Palacios-Navarro G., Sebastián-Domingo J.J. (2019). The effect of self-efficacy expectations in the adherence to a gluten free diet in celiac disease. Psychol. Health.

[28] Bandura A. (1997). Self Efficacy: The Exercise of Control.

[29] Sverker A., Hensing G., Hallert C. (2005). ‘Controlled by food’–lived experiences of coeliac disease. J. Hum. Nutr. Diet..

[30] Sverker A., Ostlund G., Hallert C., Hensing G. (2007). Sharing life with a gluten-intolerant person--the perspective of close relatives. J. Hum. Nutr. Diet..

[31] Sverker A., Ostlund G., Hallert C., Hensing G. (2009). ’I lose all these hours...’—Exploring gender and consequences of dilemmas experienced in everyday life with coeliac disease. Scand. J. Caring Sci..

[32] Casellas F., Rodrigo L., Molina-Infante J., Vivas S., Lucendo A.J., Rosinach M., Dueñas C., Fernández-Bañares F., López-Vivancos J. (2013). Transcultural adaptation and validation of the Celiac Disease Quality of Life (CD-QOL) survey, a specific questionnaire to measure quality of life in patients with celiac disease. Rev. Esp. Enferm. Dig..

[33] Rodríguez-Almagro J., Bacigalupe G., Ruiz M.C.S., González J.S., Martínez A.H. (2016). Aspectos psicosociales de la enfermedad celíaca en España: Una vida libre de gluten. Rev. Nutr..

[34] Rodríguez-Almagro J., Hernández-Martínez A., Lucendo A.J., Casellas F., Solano-Ruiz M.C., Siles-González J. (2016). Health-related quality of life and determinant factors in celiac disease: A population-based analysis of adult patients in Spain. Rev. Esp. Enferm. Dig..

[35] Leffler D., Dennis M., Edwards George J.B., Jamma S., Magge S., Cook E.F., Schuppan D., Kelly C.P. (2009). A Simple Validated Gluten-Free Diet Adherence Survey for Adults with Celiac Disease. Clin. Gastroenterol. Hepatol..

[36] Baessler J., Schwarzer R. (1996). Evaluación de la autoeficacia: Adaptación española de la Escala de Autoeficacia general. Ansiedad Estrés.

[37] Child D. (2006). The Essentials of Factor Analysis.

[38] Hicks T., McFrazier M. (2014). College Student Self-Efficacy Research Studies.

[39] Fueyo-Díaz R., Magallón-Botaya R., Gascón-Santos S., Asensio-Martínez Á., Palacios-Navarro G., Sebastián-Domingo J.J. (2018). Development and Validation of a Specific Self-Efficacy Scale in Adherence to a Gluten-Free Diet. Front. Psychol..

[40] Ware J.E., Maruish M.E., Turner-Bowker D.M., Sundaram M., Gandek B. (2009). User’s Manual for the SF-12v2 Health Survey.

[41] FACE-Federación de Asociaciones de Celíacos de España (2015). Lista de Alimentos Aptos para Celíacos, 2014–2015.

[42] Fueyo Díaz R., Gascón Santos S., Magallón Botaya R. (2016). Caracterización de la Población Celíaca en Aragón: Aspectos Psicosociales de la Adherencia A la Dieta sin Gluten.

[43] Butterworth J. (2004). Factors relating to compliance with a gluten-free diet in patients with coeliac disease: Comparison of white Caucasian and South Asian patients. Clin. Nutr..

[44] Dowd A.J., Jung M.E., Chen M.Y., Beauchamp M.R. (2016). Prediction of adherence to a gluten-free diet using protection motivation theory among adults with coeliac disease. J. Hum. Nutr. Diet..

[45] Kurppa K., Lauronen O., Collin P., Ukkola A., Laurila K., Huhtala H., Mäki M., Kaukinen K. (2012). Factors Associated with Dietary Adherence in Celiac Disease: A Nationwide Study. Digestion.

[46] Leffler D.A., Edwards George J.B., Dennis M., Cook E.F., Schuppan D., Kelly C.P. (2007). A prospective comparative study of five measures of gluten-free diet adherence in adults with coeliac disease: Measures of gluten-free diet adherence. Aliment. Pharmacol. Ther..

[47] Ludvigsson J.F., Bai J.C., Biagi F., Card T.R., Ciacci C., Ciclitira P.J., Green P.H.R., Hadjivassiliou M., Holdoway A., van Heel D.A. (2014). Diagnosis and management of adult coeliac disease: Guidelines from the British Society of Gastroenterology. Gut.

[48] Comino I., Real A., Vivas S., Síglez M.Á., Caminero A., Nistal E., Casqueiro J., Rodríguez-Herrera A., Cebolla A., Sousa C. (2012). Monitoring of gluten-free diet compliance in celiac patients by assessment of gliadin 33-mer equivalent epitopes in feces. Am. J. Clin. Nutr..

[49] de Lourdes Moreno M., Cebolla Á., Muñoz-Suano A., Carrillo-Carrion C., Comino I., Pizarro Á., León F., Rodríguez-Herrera A., Sousa C. (2017). Detection of gluten immunogenic peptides in the urine of patients with coeliac disease reveals transgressions in the gluten-free diet and incomplete mucosal healing. Gut.

[50] Luszczynska A., Gutiérez-Doña B., Schwarzer R. (2005). General self-efficacy in various domains of human functioning: Evidence from five countries. Int. J. Psychol..

[51] Luszczynska A., Scholz U., Schwarzer R. (2005). The General Self-Efficacy Scale: Multicultural Validation Studies. J. Psychol..

[52] Scholz U., Gutiérrez Doña B., Sud S., Schwarzer R. (2002). Is General Self-Efficacy a Universal Construct?. Eur. J. Psychol. Assess..

[53] Bellini A., Zanchi C., Martelossi S., Di Leo G., Not T., Ventura A. (2011). Compliance with the gluten-free diet: The role of locus of control in celiac disease. J. Pediatr..

[54] Kurppa K., Collin P., Mäki M., Kaukinen K. (2011). Celiac disease and health-related quality of life. Expert Rev. Gastroenterol. Hepatol..

[55] Casellas F., Rodrigo L., Lucendo A.J., Fernández-Bañares F., Molina-Infante J., Vivas S., Rosinach M., Dueñas C., López-Vivancos J. (2015). Benefit on health-related quality of life of adherence to gluten-free diet in adult patients with celiac disease. Rev. Esp. Enferm. Dig. Organo Soc. Esp. Patol. Dig..

[56] Zingone F., Iavarone A., Tortora R., Imperatore N., Pellegrini L., Russo T., Dorn S.D., Ciacci C. (2013). The Italian translation of the Celiac Disease-specific Quality of Life Scale in celiac patients on gluten free diet. Dig. Liver Dis..

